# Activation of the IL-17/TRAF6/NF-κB pathway is implicated in Aβ-induced neurotoxicity

**DOI:** 10.1186/s12868-023-00782-8

**Published:** 2023-02-23

**Authors:** Yulan Liu, Yang Meng, Chenliang Zhou, Juanjuan Yan, Cuiping Guo, Weiguo Dong

**Affiliations:** 1 Department of Critical Care Medicine, Renmin Hospital of Wuhan University, Wuhan, China; 2 Department of Gastroenterology, Renmin Hospital of Wuhan University, Wuhan, China; 3 Central Laboratory, Renmin Hospital of Wuhan University, Wuhan, China; 4 Department of Gastrointestinal Surgery II, Renmin Hospital of Wuhan University, Wuhan, China

**Keywords:** IL-17, Aβ, IL-17Ab, Synaptic dysfunction, Cognitive decline

## Abstract

**Background:**

Neuroinflammation plays a critical role in amyloid-β (Aβ) pathophysiology. The cytokine interleukin-17A (IL-17) is involved in the learning and memory process in the central nervous system, and its level was reported to be increased in Alzheimer's disease (AD) brains, while the effect of IL-17 on the course of Aβ has not been well defined.

**Methods:**

Here, we used APP/PS1 mice to detect the IL-17 expression level. Primary hippocampal neurons were treated with IL-17, and immunofluorescence was used to investigate whether IL-17 induced neuronal damage. At the same time, male C57BL**/**6 mice were injected with Aβ_42_ to mimic the Aβ model. Then, IL-17 neutralizing antibody (IL-17Ab) was injected into the lateral ventricle, and the open-field test, novel objective recognition test, and fear conditioning test were used to detect cognitive function. Long-term potentiation (LTP) was used to assess synaptic plasticity, molecular biology technology was used to assess the IL-17/TRAF6/NF-κB pathway, and ELISA was used to detect inflammatory factors.

**Results:**

Altogether, we found that IL-17 was increased in APP/PS1 mice and induced neural damage by administration to primary hippocampal neurons. Interestingly, using Aβ_42_ mice, the results showed that the level of IL-17 was increased in Aβ_42_ model mice, and IL-17Ab could ameliorate Aβ-induced neurotoxicity and cognitive decline in 10 C57BL/6 mice by downregulating the TRAF6/NF-κB pathway.

**Conclusion:**

These findings highlight the pathogenic role of IL-17 in Aβ-induced synaptic dysfunction and cognitive deficits. Inhibition of IL-17 could ameliorate Aβ-induced neurotoxicity and cognitive decline in C57BL/6 mice by downregulating the TRAF6/NF-κB pathway, which provides new clues for the mechanism of Aβ-induced cognitive impairments.

**Supplementary Information:**

The online version contains supplementary material available at 10.1186/s12868-023-00782-8.

## Introduction

Alzheimer’s disease (AD) is the most common type of dementia [1–4]. Amyloid beta (Aβ) is a hallmark of Alzheimer’s disease, and its accumulation in the brain is thought to play a key role in the molecular pathology of AD [5–7], Aβ is considered to have the strongest toxicity to synaptic transmission, neuronal maturation, and cognitive function [8, 9]. Neuroinflammation plays a critical role in Aβ pathophysiology [10, 11], but its etiopathogenesis is still unclear.

The interleukin 17 (IL-17) family of cytokines contains 6 structurally related cytokines, IL-17A through IL-17F. Although less is known about IL-17B–F, IL-17A (the prototypical member of this family, commonly known as IL-17) has received much attention for its proinflammatory role [12]. IL-17 is a proinflammatory cytokine produced by various types of cells, including CD4 T cells, which are categorized as a new subset called Th17 cells, acting on its specific receptor (IL-17R), which is highly expressed in the CA1 region of the hippocampus [13, 14]. After binding to IL-17, IL-17R recruits the NF-κB activator (ACT1) with the same domain through its SEFIR domain. ACT1 in turn recruits tumor necrosis factor receptor-associated factor 6 (TRAF-6), which is an adapter protein that mediates a wide array of protein–protein interactions via its TRAF domain and a RING finger domain that possesses non-conventional E3 ubiquitin ligase activity. TRAF6 was identified as a mediator of interleukin-1 receptor (IL-1R)-mediated activation of NF-κB, which plays a vital role by regulating certain functions such as neuronal plasticity and neuronal growth [15–17]. It is interesting to note that plasma IL-17 levels and neocortical Aβ load have been identified as biomarkers for AD diagnosis [18, 19]. In addition, it was reported that IL-17 triggers the onset of cognitive and synaptic deficits in the early stages of Alzheimer's disease [20]. Here, we found that IL-17 was increased in APP/PS1 mice, but the effect of IL-17 on the course of Aβ was unclear. Therefore, we further wanted to investigate whether IL17 is involved in Aβ neurotoxicity.

In our study, we investigated whether IL-17 was involved in Aβ-induced neurotoxicity and cognitive impairment. In addition, we explored its potential pathogenesis.

## Materials and methods

### Ethics statement

All methods were carried out in accordance with relevant ARRIVE guidelines (also available at https://www.arriveguidelines.org). All methods were approved by the Animal Care Committees of the Ethics Committee of Renmin Hospital, Wuhan University (IACUC Issue No. WDRY2018-K033).

### Animals and reagents

Male C57BL**/**6 mice (2 months old, 20 ± 2 g) were purchased from the Center for Animal Experiment of Wuhan University. Six-month-old male APP/PS1 mice (APPswe, PSEN1dE9 and 85Dbo/MmJNju mice) were purchased from the Model Animal Research Center of Nanjing University (Nanjing, China). The animals were housed in the Experimental Animal Center of Renmin Hospital of Wuhan University under standard laboratory conditions: natural lighting for 12 h then total darkness for another 12 h with water and ad libitum food. Mice were randomly divided into three groups: the control group, the Aβ_42_ group and the Aβ_42_ + IL-17Ab group.

### Enzyme-linked immunosorbent assays (ELISA)

The mice were first anesthetized with 2–3% isoflurane. After anesthesia, the neck was quickly severed, and the mouse’s head was placed on an ice cube to quickly extract the hippocampal tissue. The hippocampi were lysed in RIPA buffer and centrifuged at 3000 × g for 10 min at 4 °C, and the supernatant was collected. An anti-mouse IL-17 ELISA kit from Elabscience Biotechnology (Wuhan, China) was used to assay hippocampal IL-17 levels according to the manufacturer’s instructions.

### Primary hippocampal neuron culture

Five mice at 17–18 days gestation were anesthetized with 2–3% isoflurane. Fetal mice were quickly removed from the uterus and sterilized with 75% alcohol. Before the start of the experiment, the ultraclean table was irradiated by ultraviolet light for 30 min, the fetal mice were quickly transferred to an ultraclean table, and the hippocampus was dissected [21, 22]. Neurons were cultured in 12-well plates coated with 100 μg**/**mL poly-D-lysine and supplemented with 2% (v**/**v) B-27 and 1** × **GlutaMAX. Neurons cultured for 9 days were used in the experiments. In the experiment, the cells were divided into different groups: the control group and the IL-17 group (recombinant IL-17, 10 ng/mL, R&D Systems, Cat# 421-M). After treatments, cells were collected and lysed in RIPA buffer for further biological detection or fixed with 4% paraformaldehyde for immunofluorescence imaging. All cell culture reagents were purchased from Thermo Fisher Scientific.

### Stereotactic surgery

The intracerebroventricular surgery was performed with 10 mice in each group as follows: anterior–posterior: −0.3 mm; mediolateral: −1 mm; dorsoventral: -2.3 mm (from bregma and dura, flat skull). After injection, the needle was kept in place for 10 min to avoid solution reflux.

Aβ_42_ (Qiangyao Biotechnology) was oligomerized according to the procedure described previously [23, 24]. In brief, Aβ_42_ was dissolved in 1% (vol/vol) DMSO and diluted in physiological saline to a final concentration of 2.0 μg**/**μL. Then, the solution was incubated at 37 °C in darkness for 1 week before use. The mice were anesthetized with isoflurane and placed in a stereotaxic apparatus. Then, the mice were injected through the brain lateral ventricle with a solution of Aβ_42_ (5 μL), and the control group was injected with sterile normal saline containing the same volume of DMSO (1%) for 7 consecutive days.

IL-17Ab (BioXCell, clone 17F3) was dissolved in saline and injected into the lateral ventricle at 1 mg/kg in 3 μL 12 h prior to Aβ_42_ injection and 6 days post-Aβ_42_ injection. The subsequent experiments were conducted 24 h after the injection of IL-17Ab.

### Western blotting

**Fig. 1:**
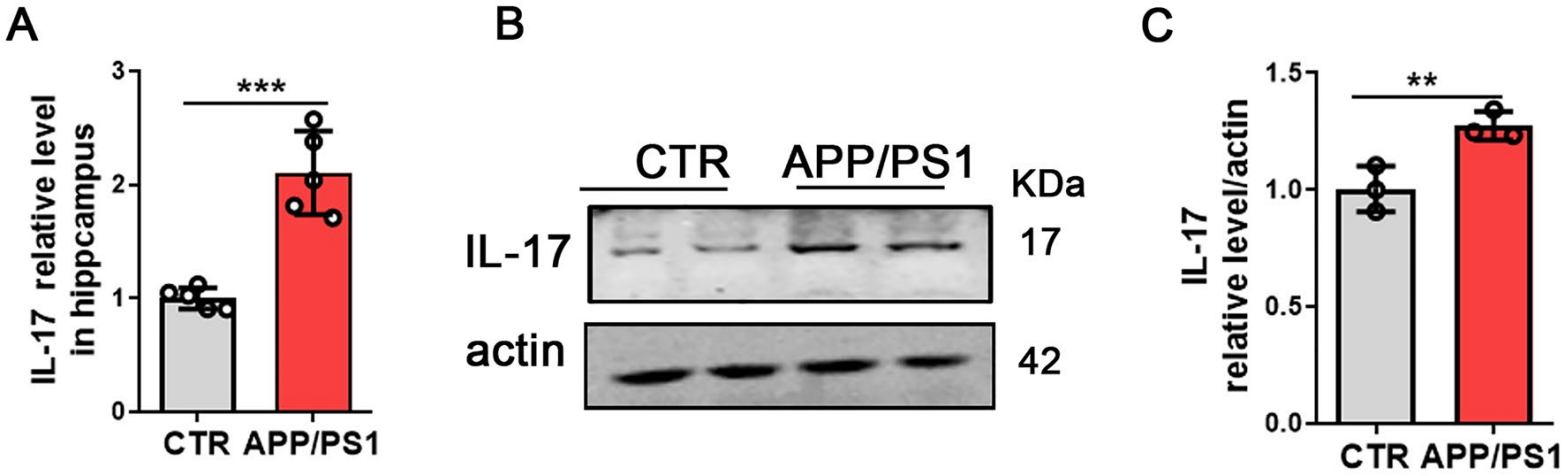
IL-17 is increased in APP/PS1 mice. Results from ELISA tests of the level of IL-17 in the hippocampus of APP/PS1 mice and control mice (**A**). N = 4. Western blotting was used to detect the IL-17 level (**B**) and quantification of IL-17 (**C**) N =3. Data are presented as the mean ± SD. *p* value significance is calculated from a T-test. **p < 0.01 and ***p < 0.001 vs the control group. For western blotting original images, please see Additional file 1, and Adobe Photoshop software (2021) was used to cut the parts of interest from the original images

The hippocampus was homogenized in a buffer (pH 7.4) containing 50 mmol**/**L Tris–HCl, 150 mmol**/**L NaCl, 10 mmol**/**L NaF, 1 mmol**/**L Na_3_VO_4_, 5 mmol**/**L EDTA, 2 mM benzamidine, and 1 mM PMSF. The supernatants were collected after centrifugation of the tissue homogenates or cell lysate at 12,000 rpm**/**min. Protein concentrations were determined with a bicinchoninic acid protein kit (Pierce, Rockford, USA). The proteins were loaded onto a 10% gel (Invitrogen, Bis–Tris), separated by electrophoresis, and then transferred to an NC membrane. The images were visualized with an Odyssey infrared imaging system (LI-COR Biosciences, USA). After extraction of animal protein, 10 μg protein was taken out of each sample for western blot experiment in Figs. 1B, 3B, 4G and 5A. Due to the slightly lower protein content of samples, 20 μg protein was taken out of each sample for western blot experiment in Fig. 5C and E. Please refer to the Additional file 1 for the original images of western blotting. For western blotting, the primary antibodies used were anti-IL-17 (Cell Signaling, #13838, 1:1000), anti-synapsin I (SYN, Millipore, AB1543, 1:1000), anti-postsynaptic density protein 95 (PSD-95, Cell Signaling, #2507, 1:1000), TRAF6 (Santa Cruz, sc-8409, 1:500), p-NF-κB p65 (Ser536) (Cell Signaling, #3033, 1:1000), anti-actin (actin, Abcam, ab6276, 1:10 000), and Lamin B1 (Abcam, ab16048, 1:1000). The protein marker was purchased from Thermo Fisher scientific in 10-180KDa, 10-250KDa and 3-200KDa.

A nuclear and cytoplasmic protein preparation kit (P1200, Pulilai) was used to separate the nuclear and cytoplasmic NF-κB p65 components according to the manufacturer’s procedures for subsequent experiments.

### Immunofluorescence staining

Hippocampal neuronal cells were rinsed with phosphate buffered saline (PBS), fixed in 4% paraformaldehyde for 8 min and permeabilized with 0.1% Triton X-100 in PBS for 30 min. After being blocked with 5% milk for 30 min, the cells were incubated with primary antibodies conjugated to Alexa Fluor^®^488 or 594 against microtubule-associated protein-2 (MAP2, Abcam, 1:250) and PSD-95 (Cell Signaling, 1:250) at 4 °C overnight. The secondary antibody was then incubated at room temperature for 1 h and rinsed in PBS three times. The nuclei were stained with DAPI (Sigma) for 5 min. Fluorescence images were obtained using a BX53 Olympus fluorescence microscope at 20 × magnification and captured using Olympus CellSens Standard software. Sholl analysis was applied to measure dendritic complexity. The length of dendritic arborization was analyzed and measured using a semi-automatized protocol via Imaris software (Bitplane, Inc.).

### Behavioral tests

#### Open-field test

The open field was used to assess the tension, anxiety, and exploration activities of the mice. In brief, 10 mice in each group were placed in sequence in a typical open field (40 × 40 cm^2^ PVC square arena with 35 cm-high walls). The inner wall of the open field reaction box was painted white, and a digital camera with a field of vision that could cover the entire open field with laboratory personnel and computer equipment located in another room was placed above the box to minimize disturbance to the animals. Movements inside the field were tracked over a 5 min period. The total distance covered and central zone crossing were tracked and measured. The chamber was sanitized with 70% ethanol after each trial.

#### Novel objective recognition test (NORT)

The 10 mice in each group were taken to the new object recognition room 24 h before the test, and then we placed the mice into a 100 cm × 100 cm × 100 cm opaque plastic container for 5 min without objects before the test. The next day, the mice reentered the container at the same starting point and were allowed for 5 min to familiarize themselves with objects A and B. After each period, the arena and objects were cleaned with 75% ethanol. Two hours after the familiarization period, the B object was replaced by the C object, and the mice were granted 5 min to explore the A object and C object. After 24 h, the C object was replaced with the D object, and the mice were also given 5 min to explore. The objects are roughly the same in height and volume but differ in shape and appearance. The exploration time for each object was recorded.

### Fear conditioning

The 10 mice in each group were placed into a square chamber (40 cm** × **40 cm × 50 cm) with white board walls, a transparent front door, and a grid floor. On the day of training, the mice were allowed to explore in an enclosed training chamber for 180 s. The mice were then exposed to a pure tone for 30 s, followed by a 2 s foot shock (0.8 mA). At 60 s after the delivery of the second shock, the mice were taken back to their home cages. Twenty-four hours later, the mice were placed in the same chamber for 3 min without foot shock for fear memory tests. The freezing time was measured using the Contextual NIR Video Fear Conditioning System (Med Associates).

### Long-term potentiation (LTP)

The intracerebroventricular LTP was performed with 3 mice in each group as follows: the mice were anesthetized with 2–3% isoflurane, and whole brains were immediately resected and soaked in ice-cold artificial cerebrospinal fluid (aCSF) saturated with 95% O_2_ and 5% CO_2_. Following sectioning at 300 μm thickness, the slices were incubated in oxygenated aCSF at 32 °C to recover for 40 min and at 20–25 °C to recover for 1 h. Then, slices were transferred to a recording chamber and submerged in aCSF perfusion. Slices were laid in a chamber with an 8 × 8 microelectrode array (Parker Technology, Beijing, China) in the bottom plane (each 50 × 50 mm in size, with an interelectrode distance of 150 μm) and kept submerged in aCSF. Signals were acquired using the MED64 System (Alpha MED Sciences, Panasonic). The field excitatory postsynaptic potentials (fEPSPs) in CA1 neurons were obtained by stimulating CA3 neurons. LTP was induced by applying three trains of high-frequency stimulation (100 Hz for 1 s, delivered 30 s apart). The LTP magnitude was quantified as the percentage change in the fEPSPs slope (10–90%) taken during the 60-min interval after LTP induction [25].

### Transmission electron microscopy (TEM)

After perfusion with fixatives, the hippocampus was dissected, and slices were approximately 150 μm thick. The slices were fixed further by immersion in 0.1 M Na-cacodylate buffer containing 2.5% glutaraldehyde for 1 h at room temperature. Postfix with 1% OsO4 in 0.1 M PBS for 2 h at room temperature. Then, dehydrate and infiltrate. Sections were photographed under a light microscope and then serially cut into semithin (2 μm thick) sections. The semithin sections were stained with 1% toluidine blue in 1% sodium borate and examined under a light microscope to locate the CA1 region. Selected semithin sections were further cut into serial ultrathin sections by using a Leica ultramicrotome. The ultrathin sections were examined under a HITACHI HT7800 TEM by an electron microscopy specialist from the Department of Ultrastructural Pathology Center, Renmin Hospital of Wuhan University. Synaptic densities were expressed as the number of synapses (identified via PSDs) per 100 μm^2^ of tissue.

### Golgi staining

The FD Rapid Golgi Staining Kit PK 401 (FD NeuroTechnologies, Inc., Columbia MO, USA) was used to measure the morphology of neuronal dendrites and dendrite spines. The 3 mice in each group were anesthetized by 2–3% isoflurane and transcardially perfused with approximately 400 mL of normal saline containing 0.5% sodium nitrite, followed by 400 mL of 4% formaldehyde solution and then 500 mL of Golgi dye solution (5% chloral hydrate, 5% potassium dichromate, and 4% formaldehyde) over 2 h. Then, the brains were dissected into 5 mm × 5 mm sections and incubated in the staining solution for 3 days and in 1% silver nitrate solution for another 3 days in the dark. Finally, the brains were sliced using a vibrating microtome (Leica, Wetzlar, Germany) at a thickness of 100 μm. Images were observed under a microscope (BX53 Olympus fluorescence microscope, Japan).

### Statistical analysis

All experiments were repeated three times. The results are expressed as the mean ± standard deviation (SD) and were analyzed using GraphPad Prism 8.0 statistical software. The normality heterogeneity of the data was tested. The differences were analyzed by the unpaired *t*-test or one-way ANOVA followed by the Bonferroni post hoc test. If the data were not normally distributed, the nonparametric test (Mann–Whitney test) was used to analyze the differences. The analyses of the quantification of the different proteins by western blot (ratio) were performed on long-transformed data. A *p* < 0.05 was considered statistically significant between groups.

## Results

### The IL-17 level is increased in APP/PS1 mice

To investigate the role of IL-17 in neuropathological changes and memory deficits associated with Aβ pathophysiology, we used the transgenic mouse model of APP/PS1 mice, a progressive model of amyloid plaques. IL-17 was increased in the hippocampus of APP/PS1 mice compared with control mice, as determined by ELISA (Fig. 1A). Furthermore, we performed western blotting, and the results showed a significant increase in the protein levels of IL-17 in the APP/PS1 mice (Fig. 1B, C), strongly supporting that the IL-17 level was increased in APP/PS1 mice.

### IL-17 induces neuronal toxicity in primary hippocampal neurons

**Fig. 2:**
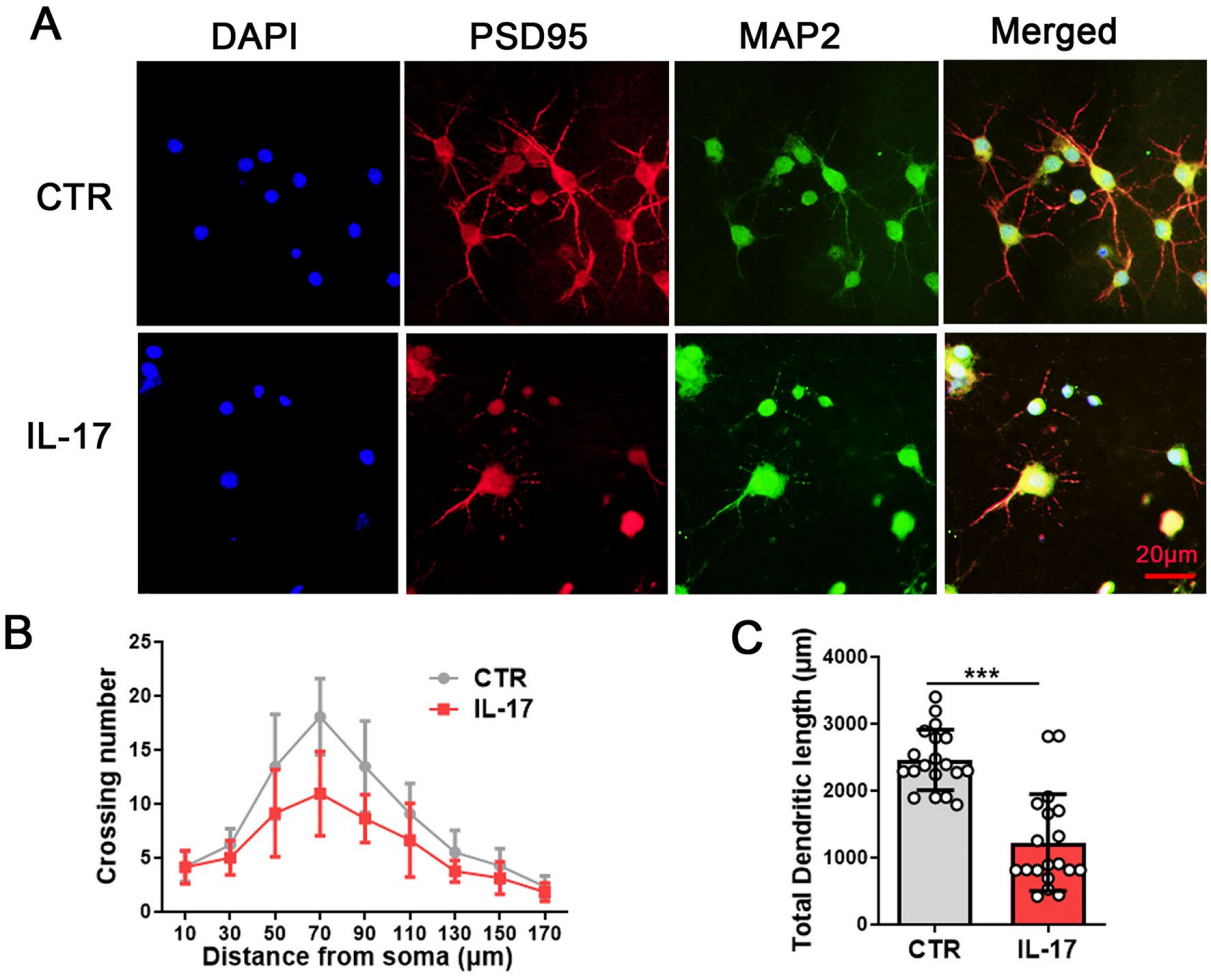
IL-17 induces neuronal toxicity in primary hippocampal neurons. Mouse primary hippocampal neurons were treated with DMSO for the control group and IL-17 for the IL-17 group for 9 d. Changes in neuronal morphology were measured by immunofluorescence staining with anti-PSD95 (red), anti-MAP2 (green) antibodies, and co-labelled with DAPI (blue). Representative images (20×magnification) after treatment (**A**), Sholl analysis (**B**), quantitative analyses of dendritic length (**C**), N = 20 hippocampal neurons. Data are presented as the mean ± SD. *p* value significance is calculated from a T-test. ***p < 0.001 vs the control group. For western blotting original images, please see Additional file 1, and Adobe Photoshop software (2021) was used to cut the parts of interest from the original images

We then investigated the effect of IL-17 on primary hippocampal neurons. The hippocampal primary neurons were divided into the control group and the IL-17 group (IL-17, 10 ng/mL). To investigate the underlying effect based on morphology, we examined the dendritic morphology of hippocampal primary neurons following treatment with IL-17 by using anti-MAP2 and PSD95 antibodies (Fig. 2A). When compared with the control, IL-17 resulted in an obviously decreased dendritic arborization complexity at all points farther than 50 μm from the cell body (Fig. 2B), as well as the total dendritic length (Fig. 2C). These findings suggest that IL-17 induced hippocampal neural damage.

### Inhibition of IL-17 alleviates Aβ_42_-induced cognitive deficits and synaptic dysfunction

**Fig. 3:**
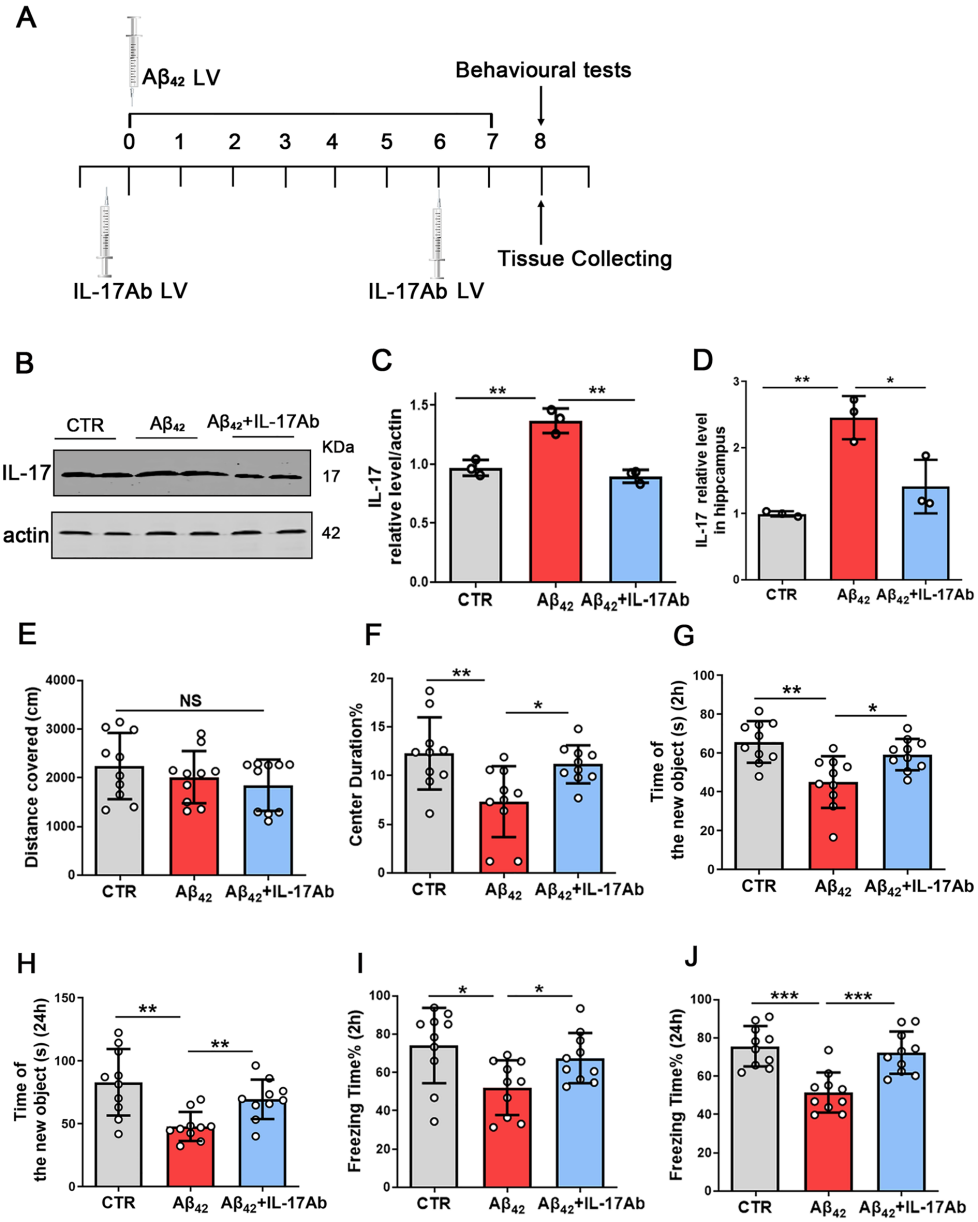
Inhibition of IL-17 alleviates Aβ_42_-induced cognitive deficits. Flow chart for the experiments (**A**). IL-17 levels were detected by western blotting using specific antibodies, and actin was used as a loading control (**B**). Intensity analysis of IL-17 levels (**C**). N = 3. ELISA to measure the levels of IL-17 (**D**), N = 3. The open-field test showed the total distance covered (**E**) and the time of center duration (**F**) of the three groups. NORT showed the time spent exploring new objects at 2 h (**G**) and 24 h (**H**). The contextual fear conditioning test determined the freezing time at 2 h (**I**) and 24 h (**J**). N = 10 for independent experiments. Data are presented as the mean ± SD. *p* value significance is calculated from a one-way ANOVA test, *p < 0.05, **p < 0.01 and ***p < 0.001 vs the Aβ_42_ group. For western blotting original images, please see Additional file 1, and Adobe Photoshop software (2021) was used to cut the parts of interest from the original images

We confirmed that the IL-17 level was increased in APP/PS1 mice and could induce hippocampal neuronal damage. Therefore, we hypothesized that IL-17 might be a target for intervention in Aβ_42_-induced neurotoxicity. To test this hypothesis, Aβ_42_ was injected into the lateral ventricle to induce the Aβ model. Then, we explored whether IL-17Ab could ameliorate Aβ_42_-induced cognitive impairment and synaptic dysfunction. We divided our experiments into three groups and provided a flow chart for the experiments (Fig. 3A). The mice in the control group were injected with saline (5 μL) in the unilateral brain ventricle, and the Aβ_42_ model group was established after injection of Aβ_42_ solution (2.0 μg/μL, 5 μL) into the unilateral brain ventricle. The Aβ_42_ + IL-17Ab group was injected with Aβ_42_ (2.0 μg/μL, 5 μL), and then IL-17Ab was injected into the lateral ventricle (1 mg/kg, 3 μL) 12 h prior to Aβ_42_ injection and the 6th day post-Aβ_42_ injection (Fig. 3A). We performed western blotting, and the results showed a significant decrease in the protein levels of IL-17 in the Aβ_42_ + IL-17Ab mice (Fig. 3B, C) compared with the Aβ_42_ mice. Furthermore, an ELISA kit for IL-17 was used to detect the IL-17 level, and the results showed that it was decreased in the hippocampus of Aβ_42_ + IL-17Ab mice (Fig. 3D).

Thirty healthy C57BL/6 mice were randomly divided into 3 groups with 10 mice in each group. Following the treatment, we performed several behavioral tests, such as the open-field test, NORT and fear conditioning test. In the open-field test, the total distance travelled showed no significant differences among the three groups (Fig. 3E), indicating that locomotion activity was not influenced by Aβ_42_ and IL-17Ab treatment, but the time spent in the center was reduced in the Aβ_42_ group compared with the control group and ameliorated by the administration of IL-17Ab (Fig. 3F), indicating that inhibition of IL-17 might reduce anxiety. The NORT showed that in the Aβ_42_ + IL-17Ab group, the curiosity of exploring new things was significantly higher when compared with the Aβ_42_ group, as the time spent exploring new objects in 2 h and 24 h tests was significantly increased (Fig. 3G, H). Then, in the fear conditioning test, the Aβ_42_ + IL-17Ab group showed a significant increase in freezing time compared with the Aβ_42_ group during the 2 h and 24 h tests (Fig. 3I, J), suggesting that inhibition of IL-17 could rescue memory function. Taken together, these data demonstrate that inhibition of IL-17 attenuates Aβ_42_-induced cognitive anxiety, social ability impairments, and learning and memory.

**Fig. 4:**
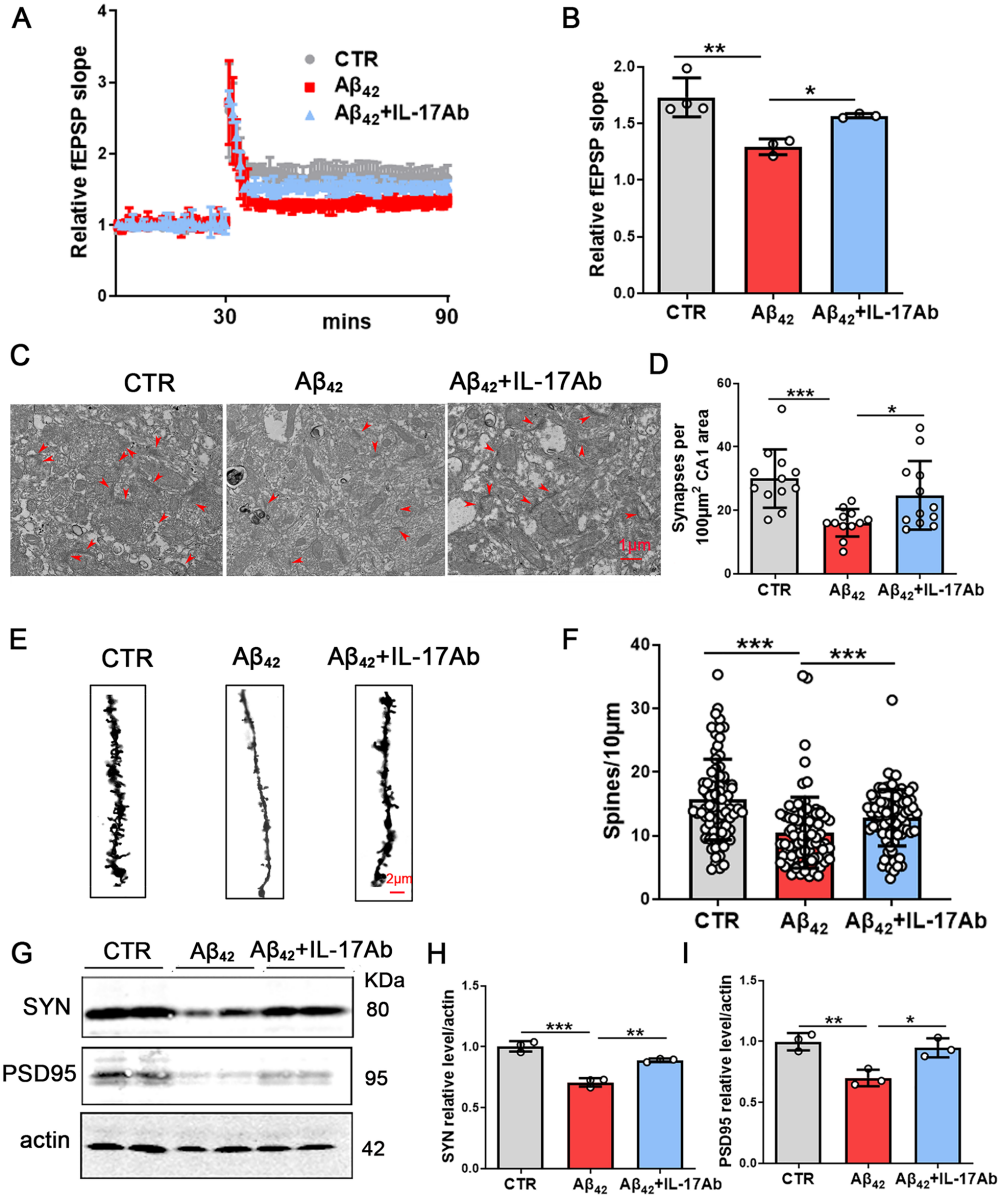
Inhibition of IL-17 alleviates Aβ_42_-induced synaptic dysfunction. Normalized CA3-CA1 fEPSPs mean slope recorded from the CA1 dendritic region in hippocampal slices (**A**). Quantitative analysis of normalized fEPSPs slopes (**B**). N = 3, brain slices per mouse were recorded. TEM images (5000×magnification) showed the structure of synapses. Red arrows indicate the structure of the presynaptic and postsynaptic membranes and the synaptic cleft (**C**). Quantitative analyses of the number of synapses (**D**), N = 12, scale bar = 1 μm. Representative images (100×magnification) of dendritic spines of neurons from the Golgi-stained hippocampus (**E**). The average spine density (mean spine number per 10-mm dendrite segment) was measured in mice (**F**), N = 90, scale bar = 2 μm. PSD95 and SYN expression levels were detected by western blotting using specific antibodies, and actin was used as a loading control (**G**). Intensity analysis of SYN (**H**) and PSD95 levels (**I**). N = 3. Data are presented as the mean ± SD. *p* value significance is calculated from a one-way ANOVA test, *p < 0.05, **p < 0.01 and ***p < 0.001 vs the Aβ_42_ group. For western blotting original images, please see Additional file 1, and Adobe Photoshop software (2021) was used to cut the parts of interest from the original images

To investigate whether IL-17 plays a role in synaptic transmission, we detected hippocampal-dependent synaptic plasticity by recording LTP. The LTP test showed that inhibition of IL-17 enhanced the slope of the fEPSPs after high-frequency stimulation (HFS) compared with that of the Aβ_42_ group (Fig. 4A, B). We also observed the number of synapses in the hippocampus with TEM, and the results showed that the number of synapses per 100 μm^2^ CA1 area increased significantly after IL-17Ab supplementation compared with that in the Aβ_42_ model group mice (Fig. 4C, D). In addition, we further examined the spine density of hippocampal neurons (Fig. 4E). Golgi staining showed a significant increase in the dendritic spine density of the Aβ_42_ + IL-17Ab mice compared with the Aβ_42_ group (Fig. 4F). We examined molecular changes in synapse-related proteins in the hippocampus. Western blotting (Fig. 4G) results showed a significant increase in the levels of SYN (Fig. 4H) and PSD95 (Fig. 4I) in the Aβ_42_ + IL-17Ab group. These results indicate that the inhibition of IL-17 alleviates Aβ_42_-induced cognitive deficits and synaptic dysfunction.

### Inhibition of IL-17 attenuates Aβ_42_-induced neuronal damage by inactivation of TRAF6-NF-κB signaling

**Fig. 5:**
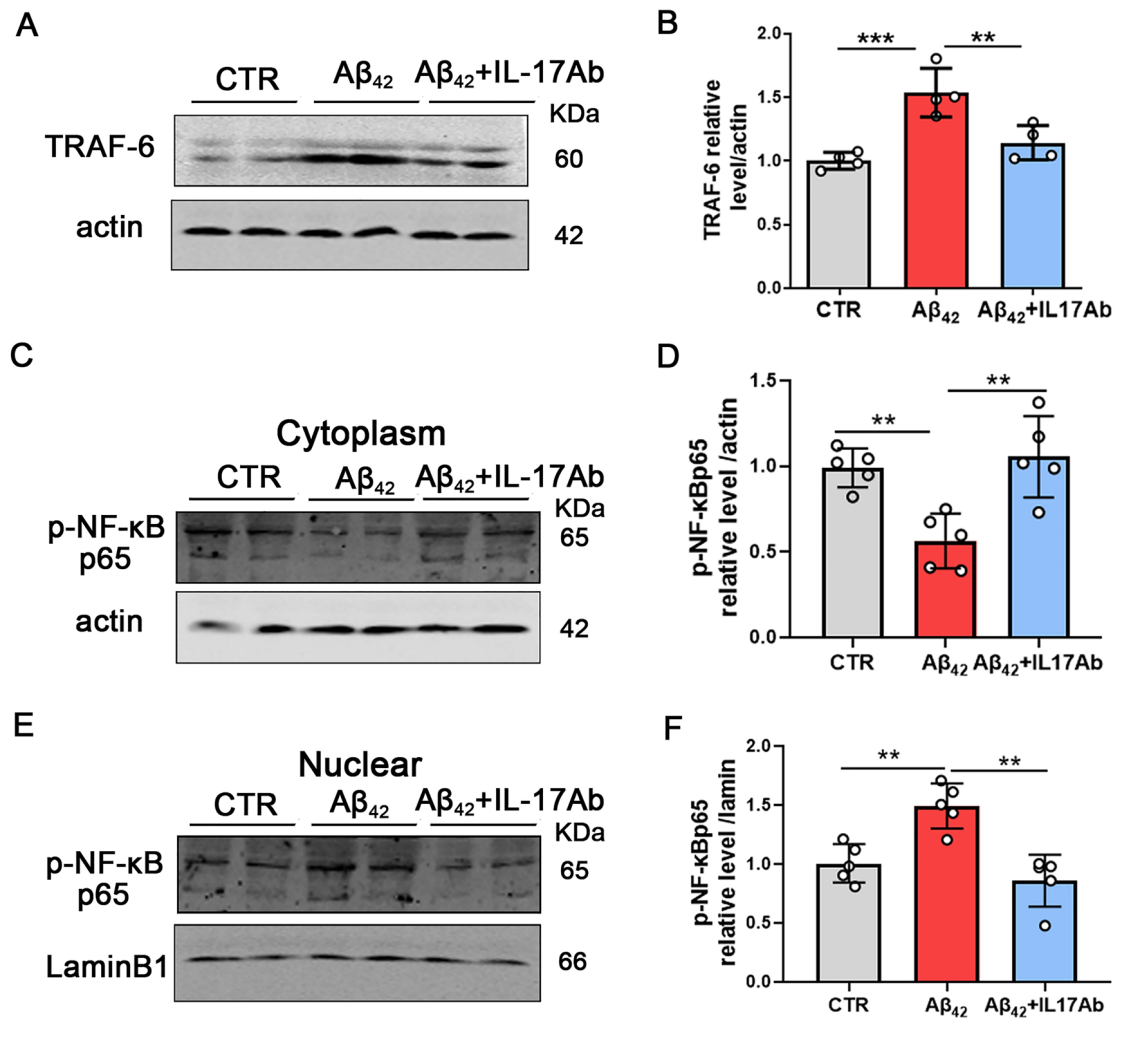
Inhibition of IL-17 attenuates Aβ_42_-induced neuronal damage by inactivation TRAF6/NF-κB signaling. TRAF-6 expression levels were detected by western blotting using specific antibodies, and actin was used as a loading control (**A**). Intensity analysis of TRAF-6 (**B**). Western blotting showed the expression of phospho-NF-κB p65 in the cytoplasm (**C**) and nucleus (**E**) in the hippocampus of mice. Intensity analysis of the phospho-NF-κB p65 levels in the cytoplasm (**D**) and nucleus (**F**). N = 5 for independent experiments. Data are presented as the mean ± SD. *p* value significance is calculated from a one-way ANOVA test, **p < 0.01 and ***p < 0.001 vs the Aβ_42_ group. For western blotting original images, please see Additional file 1, and Adobe Photoshop software (2021) was used to cut the parts of interest from the original images

To explore the mechanism of the restorative effect of IL-17Ab on learning and memory in Aβ_42_ + IL-17Ab mice, we performed western blotting to detect the levels of TRAF6. The results showed that the level of TRAF6 was increased in Aβ_42_ model mice compared with control mice, and IL-17Ab decreased the levels of TRAF6 compared with the Aβ_42_ group (Fig. 5A, B). Therefore, to explore NF-κB activation, we separated cytosolic and nuclear proteins, and western blotting results showed that Aβ_42_ treatment upregulated the translocation of p65 from the cytoplasm to the nucleus (Fig. 5C-F). IL-17Ab obviously decreased Aβ_42_-induced nuclear translocation of phospho-NF-κB p65 (Ser536) (Fig. 5C-F). Thus, these results suggest that IL-17Ab attenuates Aβ_42_-induced neuronal damage by inactivating TRAF6/NF-κB signaling.

## Discussion

Neuroinflammation plays a critical role in the pathophysiology of Aβ-induced neurotoxicity and cognitive decline [26–28]. In the present study, we found that IL-17 was increased in APP/PS1 mice, and we showed that IL-17 is involved in Aβ-induced neurotoxicity.

IL-17 is a proinflammatory cytokine produced by various types of cells, including CD4 T cells, which are categorized as a new subset called Th17 cells [29]. Th17 cells are the main cellular mediators responsible for immune-mediated damage that polarize to the site of inflammation in the presence of noxious or inflammatory stimuli [29, 30]. However, the effect of the cytokine IL-17 remains poorly understood, and very little is known about its pathophysiological role in the regions of the CNS usually compromised in disease [31]. To investigate the role of IL-17 in neuropathological changes and memory deficits, hippocampal primary neurons were treated with IL-17 (10 ng/mL), and the dendritic morphology of hippocampal primary neurons was stained using anti-MAP2 and PSD95 antibodies. The results showed that when compared with the control, IL-17 resulted in an obviously decreased dendritic arborization complexity at all points farther than 50 μm from the cell body, as well as the total dendritic length. These findings suggest that IL-17 induced hippocampal neural damage.

Therefore, we hypothesized that IL-17 might be a target for intervention in Aβ_42_-induced neurotoxicity. To test this hypothesis, we explored whether IL-17Ab could ameliorate Aβ_42_-induced cognitive impairment and synaptic dysfunction. Interestingly, using Aβ_42_ mice, the results showed that the level of IL-17 was increased in Aβ_42_ model mice, and IL-17Ab ameliorated Aβ-induced neurotoxicity and cognitive decline in C57BL/6 mice.

Then, we explored the mechanism of the restorative effect of IL-17Ab on learning and memory. Mechanistically, we show that IL-17Ab ameliorates neuronal damage in Aβ_42_ model mice, which is mediated by the inhibition of IL-17 signaling. The cellular levels of TRAF6 and nuclear phospho-NF-κB p65 (Ser536) were elevated in Aβ_42_ model mice, and administration of IL-17Ab reduced these levels. IL-17 is reported to activate NF-κB signaling through IL-17R by recruiting TRAF6. Our study showed that IL-17Ab may inactivate NF-κB and reduce its translocation from the cytoplasm to the nucleus. NF-κB signaling in the central neuron system plays a vital role by regulating certain functions, such as neuronal plasticity and neuronal growth [32], and the inhibition of NF-κB is a novel target in Alzheimer's disease therapy [33]. IL-17A bound ACT1 to IL-17-RA allows incorporation of the TRAF-6 adaptor protein into the complex, which in turn leads to activation of inhibitory κB kinase, liberating the transcription factor nuclear factor κB (NF-κB). Our results showed that Aβ_42_ treatment upregulated the translocation of p65 from the cytoplasm to the nucleus. IL-17Ab obviously decreased Aβ_42_-induced nuclear phospho-NF-κB p65 translocation. Thus, these results imply that IL-17 induces neuronal damage by activating the IL-17/TRAF6/NF-κB pathway.

The key finding in our study was that Aβ mediated neurotoxicity via the inflammatory factor IL-17. Our results showed that inhibition of IL-17 could reverse Aβ-induced neurotoxicity. In the future, we will apply this basic research to the clinical application of IL-17 inhibition to improve cognitive impairment in patients with clinical Alzheimer's disease.

## Conclusion

**Fig. 6:**
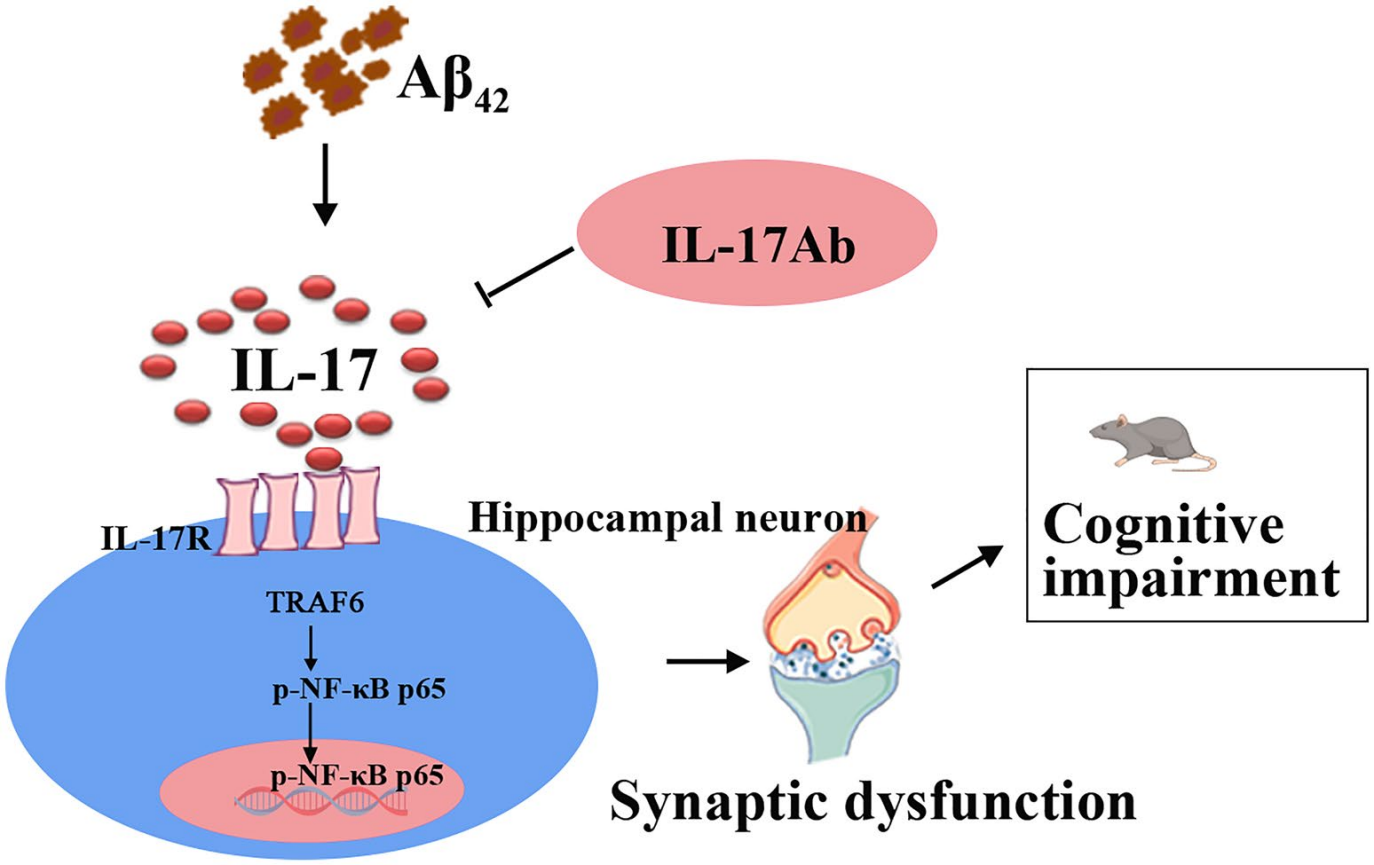
Schematic diagram of the hypothesis that activation of the IL-17/TRAF6/NF-κB pathway is implicated in Aβ-induced neurotoxicity. The level of IL-17 was increased in Aβ_42_ model mice, and IL-17Ab ameliorated Aβ-induced neurotoxicity and cognitive impairments in C57BL/6 mice by downregulating the TRAF6/NF-κB pathway

Conclusively, we have described the pathogenic role of IL-17 in Aβ-induced synaptic dysfunction and cognitive deficits. Using Aβ_42_ mice, the results showed that the level of IL-17 was increased in Aβ_42_ model mice, and IL-17Ab could ameliorate Aβ-induced neurotoxicity and cognitive decline in C57BL/6 mice by downregulating the TRAF6/NF-κB pathway (Fig. 6). Overall, this study provides new clues for the mechanism of Aβ-induced cognitive impairments.

### Supplementary Information

**Additional file 1.** Figure S1B, S3B, S4G and S5.

## Data Availability

The datasets used and/or analyzed during the present study are available from the corresponding author on reasonable request.

## Abbreviations

AD: Alzheimer’s disease
Aβ: Amyloid-β
CTR: Control
DMSO: Dimethyl sulfoxide
ELISA: Enzyme-linked immunosorbent assay
fEPSPs: Field excitatory postsynaptic potentials
IL-17A: Interleukin-17A
IL-17Ab: Interleukin-17A antibody
LTP: Long-term potentiation
NF-κB: Transcription factor nuclear factor κB
NORT: Novel objective recognition test
PSD-95: Postsynaptic density protein 95
SYN: Synapsin I
TEM: Transmission electron microscopy
TRAF-6: Tumor necrosis factor receptor-associated factor 6
PBS: Phosphate buffered saline
aCSF: Artificial cerebrospinal fluid
Fig: Figure
