# Correction: Activation of the IL-17/TRAF6/NF-κB pathway is implicated in Aβ-induced neurotoxicity

**DOI:** 10.1186/s12868-023-00804-5

**Published:** 2023-08-24

**Authors:** Yulan Liu, Yang Meng, Chenliang Zhou, Juanjuan Yan, Cuiping Guo, Weiguo Dong

**Affiliations:** 1 Department of Critical Care Medicine, Renmin Hospital of Wuhan University, Wuhan, China; 2 Department of Gastroenterology, Renmin Hospital of Wuhan University, Wuhan, China; 3 Central Laboratory, Renmin Hospital of Wuhan University, Wuhan, China; 4 Department of Gastrointestinal Surgery II, Renmin Hospital of Wuhan University, Wuhan, China

**Correction:** ***BMC Neurosci *24**, **14 (2023)**

10.1186/s12868-023-00782-8

Following the publication of the original article [1], the editors reported that there were some inconsistencies in the use of English. The authors provided an edited version of the manuscript.

The original article [1] has been updated. The content was not affected by this correction.
