# Supplementary figures and images for: Activation of the IL-17/TRAF6/NF-κB pathway is implicated in Aβ-induced neurotoxicity
Source: BMC Neurosci. 2023 Feb 23;24:14. doi: 10.1186/s12868-023-00782-8 (PMC9951515; doi:10.1186/s12868-023-00782-8)

**Figure 1B**

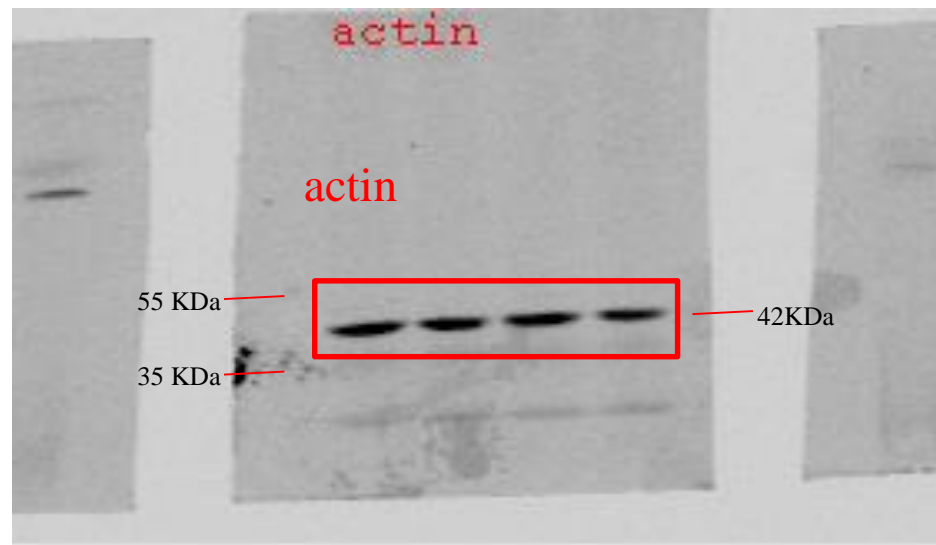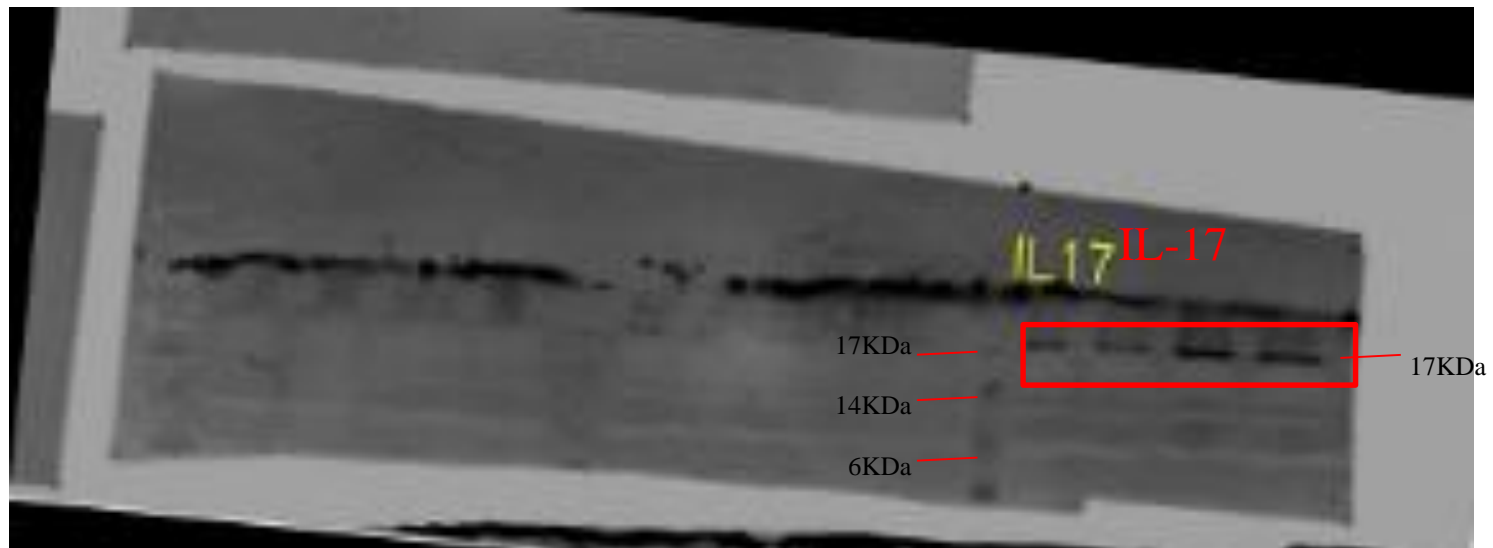

**Figure 3B**

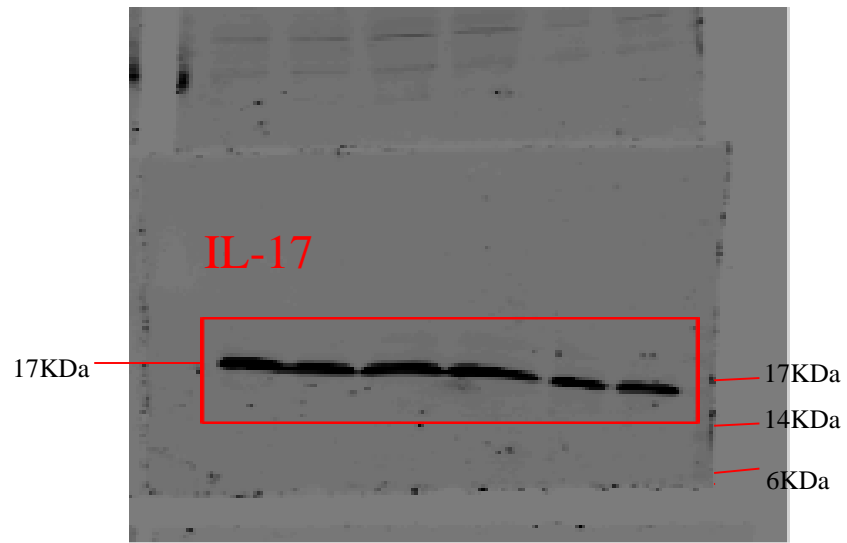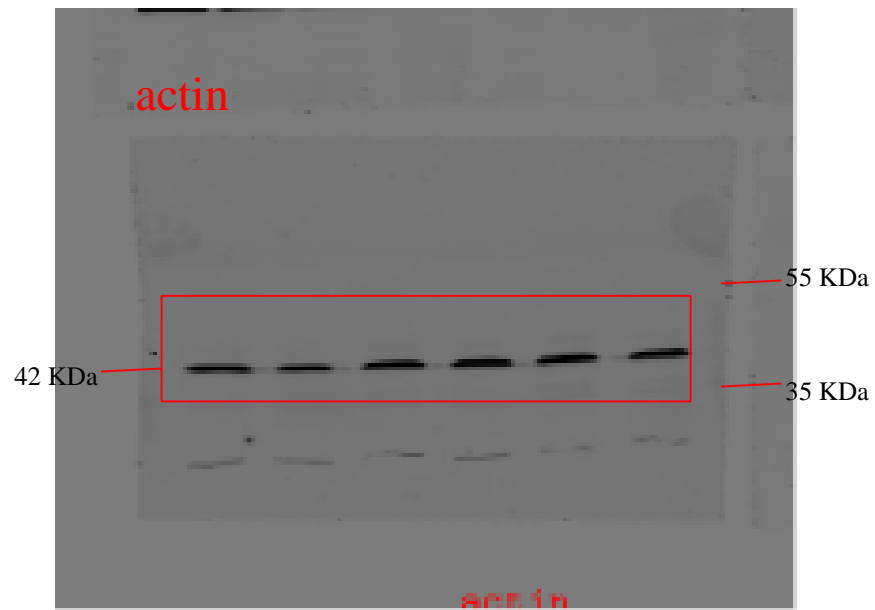

**Figure 4G**

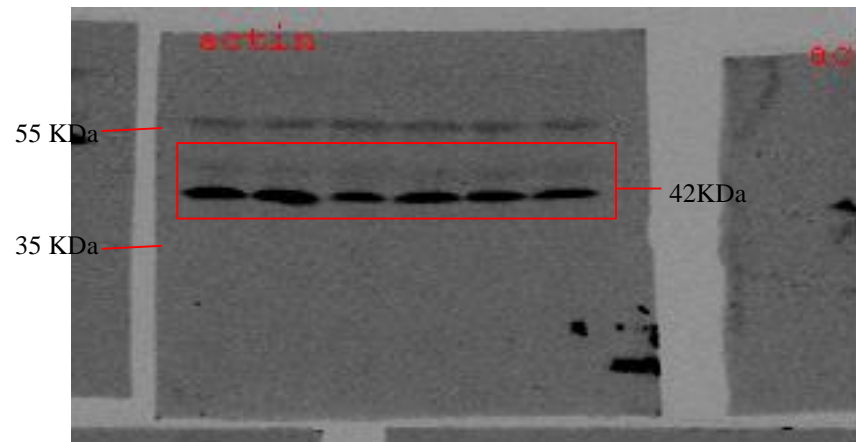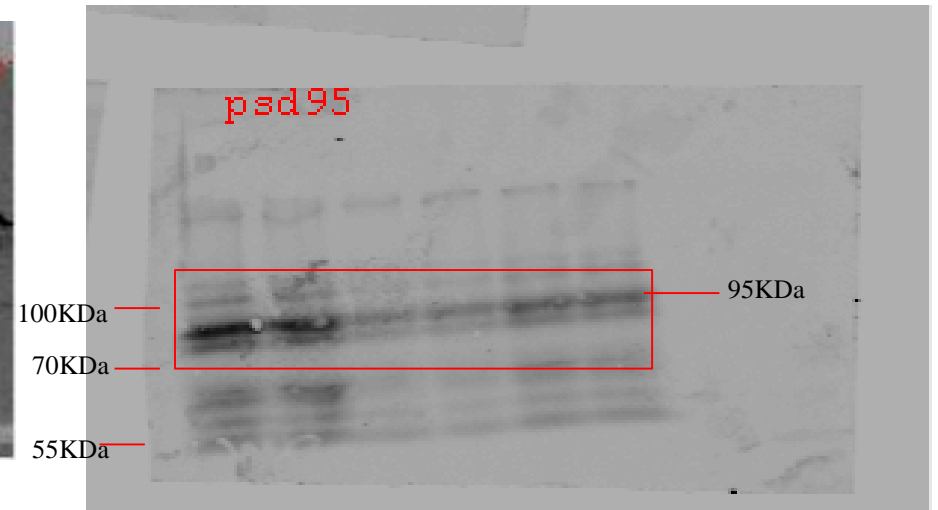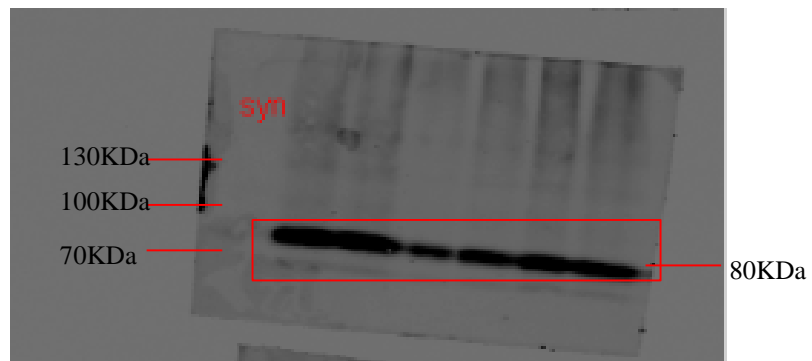

**Figure 5**

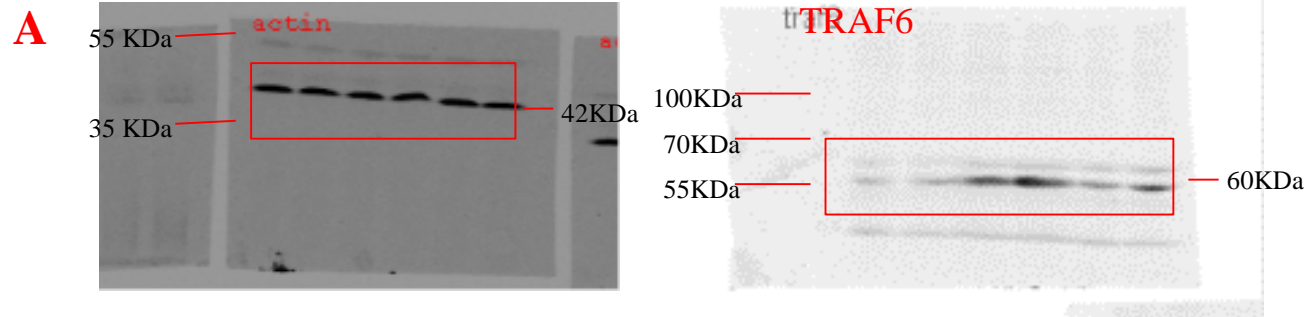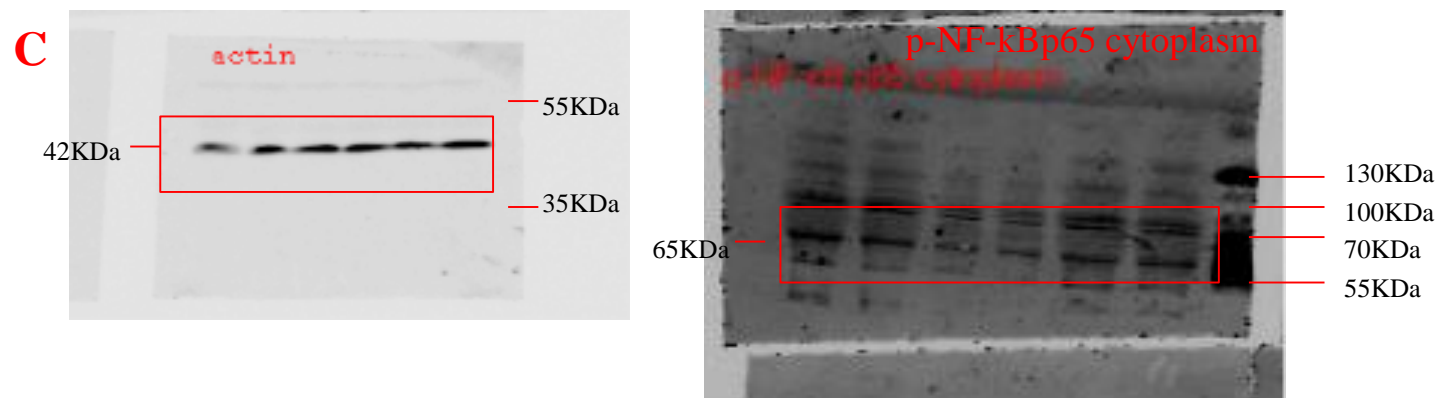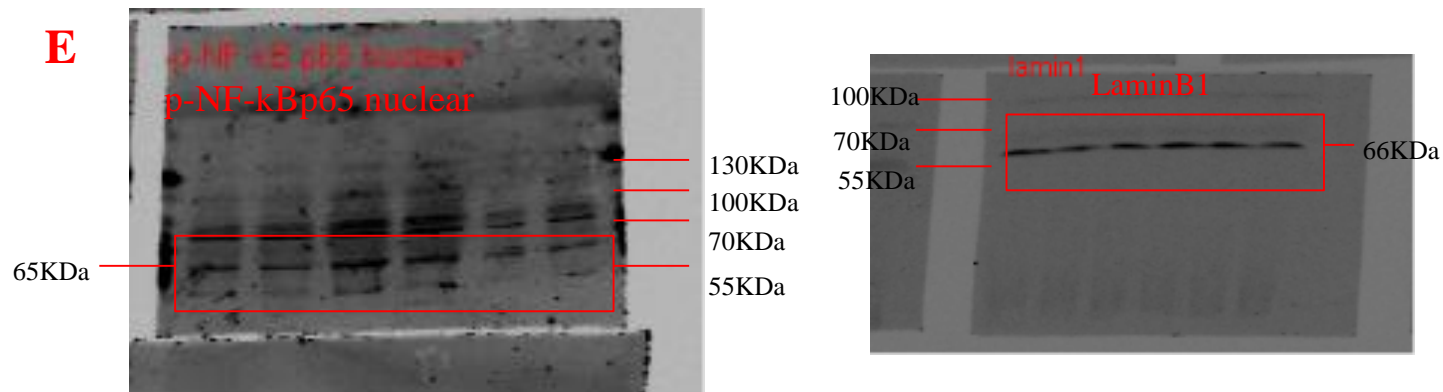

Supplement: Supplementary file 1 — Additional file 1. Figure S1B, S3B, S4G and S5. [file 12868_2023_782_MOESM1_ESM.pdf]
